# Influence of the HLA-Cw6 Allele and *IFIH1/MDA5* Gene Variants on the Cardiometabolic Risk Profile of Patients with Psoriatic Disease

**DOI:** 10.3390/jcm13030845

**Published:** 2024-02-01

**Authors:** Rubén Queiro, Ignacio Braña, Estefanía Pardo, Marta Loredo, Stefanie Burger, Pablo González del Pozo, Paula Alvarez, Eva Fernández-Bretón, Pablo Coto, Eliecer Coto

**Affiliations:** 1 Rheumatology Division, Hospital Universitario Central de Asturias (HUCA), 33011 Oviedo, Spain; i.brana.abascal@hotmail.es (I.B.); estefaniapardoc@gmail.com (E.P.); mloredomart@gmail.com (M.L.); stefanie.nam@gmail.com (S.B.); pgd795@hotmail.com (P.G.d.P.); paulalvarez992@gmail.com (P.A.); 2 Translational Immunology Division, Health Research Institute of Asturias, 33011 Oviedo, Spain; 3 Department of Medicine, Faculty of Medicine, Oviedo University, 33011 Oviedo, Spain; eliecer.coto@sespa.es; 4 Biostatistics and Epidemiology Platform, Health Research Institute of Asturias, 33011 Oviedo, Spain; eva.fbreton@ispasturias.es; 5 Dermatology Division, Hospital Vital Alvarez Buylla, 33611 Mieres, Spain; pablocotosegura@gmail.com; 6 Molecular Genetic Unit, Hospital Universitario Central Asturias (HUCA), 33011 Oviedo, Spain

**Keywords:** HLA-Cw6, *IFIH1/MDA5* gene, psoriatic arthritis, psoriasis, cardiovascular risk

## Abstract

Background: Cardiovascular comorbidity is a common companion of psoriasis and psoriatic arthritis (PsA). Recently, a significant link has been found between the HLA-Cw6 allele and a better cardiometabolic profile in these patients. We aimed to check this finding in our setting. Methods: A cross-sectional observational study (n: 572 psoriasis patients, 30% with PsA) was conducted. Different study variables were collected in detail, as well as classic cardiometabolic risk factors. The distribution of the HLA-Cw6 allele and the *IFIH1/MDA5* gene variants previously linked to disease risk were determined in the study cohort and stratified according to the cardiometabolic comorbidity. Linear and logistic regression models were constructed to analyze these associations. Results: The study cohort included 309 men and 263 women, with a mean age of 46.7 years (SD 14.5) and a mean disease duration of 19.4 years (SD 14.8). We confirmed the known association between HLA-Cw6 and type I psoriasis (familial, severe, and early onset). Psoriasis severity (OR: 2.14), female sex (OR: 1.63), and the *IFIH1/MDA5* rs1990760 TT genotype (OR: 1.62) were significantly related to PsA, while HLA-Cw6 was protective (OR: 0.65). HLA-Cw6 carriers showed a lower waist perimeter, lower BMI, and lower risk of both hypertension (OR: 0.52, *p* < 0.001) and diabetes (OR: 0.36, *p* < 0.001), but these findings were no longer apparent upon adjusting the regression models. No *IFIH1/MDA5* gene variant was associated with any cardiometabolic risk factor. Conclusions: The influence of HLA-Cw6 on the cardiometabolic risk profile of psoriatic patients seems to be explained by other factors (age, sex, duration of the disease or arthritis) and not by this biomarker itself.

## 1. Introduction

Psoriasis and psoriatic arthritis (PsA) are complex entities with a high clinical heterogeneity in which skin/nail manifestations overlap, to varying degrees, with musculoskeletal manifestations. Thus, it is not uncommon for patients to present different combinations of peripheral and axial arthritis, dactylitis and/or enthesitis over time, in addition to cutaneous manifestations. Along with this, other manifestations may occur such as uveitis or inflammatory bowel disease, as well as a wide variety of comorbidities, mainly cardiometabolic [1]. This clinical complexity has supported the modern concept of psoriatic disease as a systemic entity that goes beyond skin and joint manifestations to encompass all the aforementioned aspects, ultimately conditioning the prognosis, sometimes gloomy, and in many cases the therapeutic approach [2].

Psoriatic disease has a variegated and complex genetic basis, where the HLA-Cw6 allele stands out, not only as the main genetic determinant of the disease, but also as one of the key alleles responsible for its varied phenotypic expression [3]. Classically, the presence or absence of this marker has been linked to specific disease endotypes. In addition to being associated with severe, early onset, familial psoriasis (type I disease), HLA-Cw6 has been associated with longer psoriasis–arthritis latency, a better response to certain therapies (ustekinumab and methotrexate), and, very recently, to a better cardiometabolic risk profile [4,5,6]. In this last sense, two independent groups of researchers have related the positivity of this biomarker to a lower risk of hypertension and less visceral adiposity [4,5]. Also, very recently, Spanish researchers have found a link between HLA-Cw6 and a lower risk of liver fibrosis in PsA [7]. In summary, it seems that carriers of this allele would have a better cardiometabolic risk profile, which has sparked renewed interest in the usefulness of routinely requesting this biomarker in the management of psoriatic disease [8].

However, studies that point to a cardiometabolic benefit of this marker are still few, and the unequal distribution of this allele in different psoriasis populations worldwide demands more studies that expand and support the HLA-Cw6-linked endotype. On the other hand, since HLA-Cw6 is associated with the early onset skin disease, it would be interesting to investigate whether other recently described genetic biomarkers of the early onset disease have the potential to be linked to the cardiometabolic risk that is characteristic of this disease [9]. In the present report, the distribution and possible connection between the HLA-Cw6 allele and *IFIH1/MDA5* genetic variants with the different cardiometabolic risk factors are analyzed in a large cohort of patients with psoriatic disease.

## 2. Materials and Methods

### 2.1. Study Population

A two-center, observational and cross-sectional study of 572 patients with psoriatic disease, of which 30% presented CASPAR criteria for PsA, was carried out. All of them were over 18 years of age and of Caucasian ancestry, residing in the Principality of Asturias (northwestern Spain, total population 1,000,000 inhabitants). The study cohort was recruited between January 2007 and August 2017 from the dermatology and rheumatology departments of two hospitals. All patients were informed of the purposes of the study and gave their informed consent to participate in this research. The anonymity of the study subjects was preserved at all times, and the rules of good clinical practice in the Declaration of Helsinki have been respected throughout the study. The study protocol was approved by the research ethics committee of the Principality of Asturias.

### 2.2. Sociodemographic and Clinical Variables

From the sociodemographic point of view, age, sex, and educational level were collected. Age at disease onset, family history of disease, type of psoriasis and its severity, PASI, nail involvement, and treatments received were also recorded. Severe disease was regarded as PASI ≥ 10. In the case of PsA, and for the purposes of this study, we simply recorded its presence according to CASPAR criteria, with subsequent diagnostic confirmation by a rheumatologist experienced in this pathology. Smoking habits and alcohol consumption were recorded. Throughout the study period, the presence/absence of high blood pressure, diabetes and its types, dyslipidemia, fatty liver disease, weight, height, BMI, and waist circumference were determined. Basal blood glucose level, total cholesterol, HDL, LDL, and triglycerides were recorded. For the purposes of the study, early disease was regarded as disease onset ≤ 40 years (n: 440), while late-onset was reserved for patients with disease onset > 40 years (n: 132).

### 2.3. Disease Risk Genotyping

All patients were genotyped for the HLA-Cw6*0602 allele (SNP rs1050414 C/G). The DNA was obtained from whole blood leukocytes, and all the individuals were genotyped for the next IFIH1 SNPs: rs1990760 (g.162267541C > T, c.2719G > A, p.Ala907Thr), rs35337543 (g.162279995C > G, c.1641 + 1G > C), and rs35744605 (g.162277580C > A, c.1879G > T, p.Glu627Ter). The three IFIH1 SNPs were determined using real-time PCR TaqMan assays (Fisher Scientific, Waltham, MA, USA): rs35337543, assay ID C_25985625_10; rs35744605, assay ID C_25982959_10; rs1990760, C_2780299_30. The quality of the genotyping method was confirmed via sequencing PCR fragments with different genotypes. We determined the population frequency of the IFIH1 variants in a total of 200 controls aged 21–75 years (50% aged ≤40 years). They were individuals from our general population without other inclusion or exclusion criteria. They were recruited with the main purpose of determining the allele/genotype frequencies of the IFIH1 SNPs in our population. These controls were genotyped for the three SNPs with the above referenced TaqMan assays [9].

### 2.4. Statistical Analysis

For the univariate descriptive analysis, the mean, standard deviation, median, minimum and maximum value of the quantitative variables, and the absolute and relative frequencies (percentages) of the categorical variables are presented. A descriptive and comparative analysis of the variables included based on sex and the HLA-Cw6 marker is also presented. To estimate the crude effect of the genetic markers on the different outcomes, simple linear regression models were constructed when the dependent variable was a quantitative variable, and simple logistic regression models when the dependent variable was categorical. To estimate the adjusted effect, multiple regression models were created (linear or logistic, as appropriate) where covariates such as age, sex, time of evolution, and the presence of arthritis were introduced. We performed a logistic regression to compare the difference in sex, severity (PASI ≥ 10), PsA, and the HLA-Cw6 positivity, stratified by age at onset. The allele and genotype frequencies between groups were compared with the chi-squared and Fisher’s exact tests. For the genetic comparisons, we considered alleles previously related to increased IFIH1 expression or function as putative risk factors (rs1990760 T, rs35337543 G, and rs35744605 C). The threshold for statistical significance was set at *p* < 0.05. Data were analyzed using R software (4.3.1 “Beagle Scouts”).

## 3. Results

### 3.1. Study Population Characteristics

The study cohort was composed of 309 men and 263 women, with a mean age of 46.7 years (SD 14.5), and a mean disease duration of 19.4 years (SD 14.8). The median PASI was 14 (min: 1, max: 49), and 294 (51.4%) patients had a PASI ≥ 10. Nail dystrophy was diagnosed in 328 patients (57.3%), while in 22 (3.8%) this aspect was not recorded in their clinical history. In 222 (38.8%) patients, nail involvement was not detected in any of the visits recorded. A family history of psoriasis and/or PsA was found in 211 patients (36.9%). Regarding the anthropometric variables, the average weight was 77.9 kg (SD 16.4) with an average BMI of 27.6 (SD 5.02), with the average waist circumference being 98 cm (min: 61, max: 138). Just over a third (34.3%) of the patients were smokers, while the median alcohol consumption according to standard drink units (SDU) was 0 (min: 0, max: 30). Of the study patients, fatty liver disease was ruled out in 405 (70.8%), confirmed in 129 (22.6%) and no information was available for 38 (6.6%). A total of 20% of the patients had hypertension and another 20% had dyslipidemia. In total, 33 patients had adverse coronary events (5.8%), 22 patients were type I diabetics (3.8%), while 45 (7.9%) were type 2. A total of 241 patients expressed the HLA-Cw6 allele (42.1%). The distribution of *IFIH1/MDA5* genetic variants within the study population has been published elsewhere [9]. A total of 440 patients had an onset age of ≤40 years compared to 132 cases at age > 40 years. Severe disease (PASI ≥ 10), family history of disease, and the presence of HLA-Cw6 were significantly more frequent in patients with onset age ≤ 40 years (*p* < 0.001). Upon multiple logistic regression analyses for the presence of PsA, we found significant associations with psoriasis severity (OR: 2.14, 95%CI: 1.46–3.16), female sex (OR: 1.63, 95%CI: 1.12–2.38), and the *IFIH1/MDA5* rs1990760 TT genotype (OR: 1.62, 95%CI: 1.11–2.37), while the HLA- Cw6 allele was protective (OR: 0.65, 95%CI: 0.44–0.95). Before inclusion in the study, the patients had undergone various therapies for their disease: half had received methotrexate, 20% had received retinoids, and a third had been exposed to phototherapy. Table 1 and Table 2 represent the distribution of the study variables stratified by sex and HLA-Cw6, respectively.

### 3.2. Relationship between Cardiometabolic Risk Factors and HLA-Cw6

Regarding waist circumference, in the crude model, subjects carrying HLA-Cw6 had an average of 2.37 cm less (95%CI: −4.82, 0.07), *p* = 0.06. This difference was lost in the model adjusted for age, sex, duration of disease, and arthritis [0.05 (−2.19, 2.29), *p* = 0.965]. Regarding BMI, in the crude model, HLA-Cw6 positives had an average of 0.81 units less (95%CI: −1.67, 0.05), *p* = 0.06, but in the model adjusted for the previous variables, this difference was no longer apparent [−0.11 (95%CI: −0.96, 0.74), *p* = 0.80]. HLA-Cw6 subjects reduced the risk of hypertension by 48% (95%CI: 20–66). However, the model adjusted for age, sex, disease duration, and the presence of arthritis demonstrated a 17% reduction, without being statistically significant (95%CI: 0.49–1.40, *p* = 0.49). Cw6 patients had a lower risk of diabetes, OR: 0.36, 95%CI: 0.19–0.63, *p* < 0.001; however, after adjustment for age, this association was not maintained, OR: 0.56, 95%CI: 0.28–1.05, *p* = 0.08. There was no relationship, in either the crude or adjusted models, between HLA-Cw6, obesity, dyslipidemia, fatty liver disease, or adverse coronary events.

### 3.3. Relationship between Cardiometabolic Factors and IFIH1/MDA5 Genetic Variants

Patients carrying the *IFIH1/MDA5* rs1990760 variant had a disease that began on average almost six years earlier than subjects without this biomarker (95%CI: −9.93, −1.87, *p* = 0.004). However, there was no association between *IFIH1/MDA5* genetic variants and cardiometabolic risk factors, neither in the crude nor in the adjusted regression models.

## 4. Discussion

In this study, we have investigated the connections between two of the genetic biomarkers most consistently linked to the risk of psoriatic disease (HLA-Cw6 and variants of the *IFIH1* gene) and the presence of the cardiometabolic comorbidity typical of this condition. We confirmed the known relationship between the HLA-Cw6 allele and type I disease (early, familial, and severe), while PsA was independently and positively associated with female sex, severe psoriasis, and the *IFIH1/MDA5* rs1990760 TT variant, but negatively with HLA-Cw6. In the unadjusted regression models, we detected a trend of HLA-Cw6 positive patients towards a better cardiometabolic profile (lower BMI, less visceral adiposity, lower risk of hypertension and lower risk of diabetes). However, in the adjusted multiple regression model, these differences were no longer apparent. No variant of the IFIH1 gene was found to be associated with cardiovascular (CV) comorbidity.

It has been known for decades that psoriatic patients carrying the HLA-Cw6 allelic variant present specific features of the disease (HLA-Cw6-linked endotype). This essentially refers to the presence of a more severe psoriasis, with a greater familial inheritance burden and an earlier age of disease onset (type I psoriasis) [3]. There are also repeated reports regarding the relationship between this allele and a better response to certain therapies [3,10], as well as links with other characteristics of psoriatic disease, such as the negative relationship with nail dystrophy [3,5], also found in our study (OR 0.40, *p* < 0.001). Although the connection between HLA-Cw6 and a lower presence of diabetes [11] is known, it has not been until very recent times (the last two years) that studies have started to appear that connect this allele with a better overall cardiometabolic risk profile [4,5,6]. In fact, at least two recent independent studies carried out in populations of European Caucasian ancestry have found associations between HLA-Cw6 and a smaller waist circumference, less visceral adiposity, and, in general, a “protective” tendency against the development of certain CV risk factors such as hypertension. Thus, in these studies, the reduction in the risk of hypertension conferred by HLA-Cw6 has been around 30% [4,5]. A study carried out in a Spanish population also found a reducing effect of HLA-Cw6 on the risk of liver fibrosis in subjects with PsA, while curiously enough, the presence of hypertension increased this risk [7]. A fourth study carried out in a Scandinavian population found less visceral adiposity in HLA-Cw6 psoriasis subjects [6]. However, although our results presented a similar trend to the aforementioned data in the unadjusted models, we could not confirm these findings in the adjustments based on age, sex, disease duration, and the presence of arthritis. Therefore, our findings seem more in line with those works that independently find a greater prevalence of cardiometabolic factors in subjects with PsA in relation to patients with psoriasis who do not develop arthritis [12,13,14]. This is especially interesting if we take into account that, as we were also able to confirm here, the associations between HLA-Cw6 and arthritis (negative association) seem to go in the opposite direction to the same relationship when analyzed in patients with psoriasis without arthritis (positive association). In other words, the possible relationship between the negativity for this allele and a greater presence of cardiometabolic comorbidity seems to be better explained by age and the presence of PsA.

What, then, could be the reason for the disparity in the results found between our work and others from the recent literature? First of all, we must consider that, for example, in the work by Douroudis et al. [5], more than half of the patients were HLA-Cw6 positive, while in our cohort it was slightly higher than 40%, and in the HUNT4 study by Solvin et al. it did not exceed 30% [6]. Furthermore, the prevalence of psoriasis in the HUNT4 study was greater than 6% of the population [6], a figure that clearly exceeds the prevalence of the disease in our latitude [15]. Another possibility is the different frequency that these comorbid factors (hypertension or diabetes, for example) may have in these studies, as well as the different duration times of the disease. Regarding this last point, the average duration of the disease in our cohort did not exceed 20 years, while in the studies by Douroudis et al. and Solvin et al., it clearly exceeded this threshold [5,6]. As our study is a punctual observation in time, we cannot rule out that with a longer follow-up period there may be a higher prevalence of some of the comorbidities analyzed, and that this, in turn, represents a relevant change in our results.

Age and disease duration turned out to be explanatory factors for some of the associations found here between the disease and cardiometabolic factors. Therefore, it seemed, a priori, interesting to include other genetic markers that have been related to the age of onset of the disease (variants in the *IFIH1/MDA5* gene) and to verify whether these variants contributed to a differential distribution of CV comorbidity factors. However, although the disease began an average of six years earlier in subjects carrying the *IFIH1/MDA5* rs1990760 variant, neither this nor any other allelic variant of the *IFIH1/MDA5* gene explained a different distribution of CV comorbid factors in our population.

In the work of Macia-Villa et al., HLA-Cw6 was protective against liver fibrosis (an advanced stage of fatty liver disease) in subjects with PsA [7], although we could not find any relationship between fatty liver disease and the different allelic variants analyzed here. In any case, it is important to highlight that the prevalence of fatty liver disease in our cohort was well below what would be expected according to the figures that have been previously published in this regard [16], so our findings at this specific point should be taken with caution.

The female population in our study had a higher frequency of arthritis and HLA-Cw6 positivity, similar to what was found by Douroudis et al. [6]. However, none of the differences between genders in the distribution of CV comorbidity factors (Table 1) could be explained by the genetic markers included in this study. In any case, it is necessary to highlight that the genetic associations reported with respect to CV risk in psoriatic disease are quite heterogeneous, which makes it difficult to have a clear position on the matter [17,18,19,20].

Therefore, what explained the presence of cardiometabolic comorbidity, regardless of HLA-Cw6 in our study? Starting with waist circumference, a surrogate marker of visceral adiposity: for each decade of age this circumference increased by 3 cm (*p* < 0.001), and women had an average of almost 10 cm less waist circumference (*p* < 0.001); evolution time, whereby individuals with a short evolution time had a lower waist circumference (*p* = 0.021); and arthritis, whereby patients who developed PsA had 3.68 cm more waist circumference than patients without arthritis (*p* = 0.003). The BMI analysis showed results in line with the above. Regarding hypertension, for each decade of age, the risk increased by 11% (*p* < 0.001), and arthritis increased this risk by 65% (*p* < 0.05). Diabetes was essentially explained by older age, whereby for each decade, this risk increased by 8% (*p* < 0.001). For each decade of age, the risk of dyslipidemia increased by 7% (*p* < 0.001), while arthritis increased this risk by 86% (*p* = 0.008). The risk of fatty liver disease increased by 2% for each decade of age (*p* = 0.02) and was reduced by 74% in women compared to men (*p* < 0.001). Adverse coronary events increased by 11% per decade (*p* < 0.001). Interestingly, the finding of a relationship between PsA and the risk of certain factors such as hypertension or dyslipidemia had also been collected by other studies [12,13,14,21,22].

In a previous study by our group, we had detected a cardiometabolic endotype linked to HLA-Cw6 [4]. However, that study was carried out for clinical phenotyping purposes (in fact, the characterization of the PsA population was much finer), while the present cohort was constructed for genotyping studies, so the collection of variables was much more selective and exhaustive (for example, in the first cohort no information on fatty liver disease was collected, while in the cohort of the present study it was). Therefore, the studies are not an extension of each other, but rather very different.

The main limiting factor of our findings is the punctual and cross-sectional nature of the observation, which logically prevents causal inferences. However, we included a substantial number of well-characterized patients with a relatively long mean follow-up period. Being a cohort designed for disease risk genotyping purposes, all variables have been carefully checked. The loss of data has also been very small. As we have mentioned previously, we do not know if with a longer follow-up observation period a greater number of comorbidities could have been detected, and, therefore, a change in the results of the present study. We have not included important aspects such as tobacco or alcohol consumption among the corrective factors of the regression models; however, we do not believe there is any connection between the genetic markers analyzed and these harmful habits. One of the important types of information that we have not been able to collect is the psychological status of the patients at the time of recruitment, since it is known that there is a close connection between the cytokines involved in the pathogenesis of the disease and the anxiety or depression so common in these patients. On the other hand, arthritis has been introduced as a dichotomous variable, without us being able to provide a finer characterization of these patients.

## 5. Conclusions

Although in this study we have not been able to confirm a relationship between HLA-Cw6 and a better cardiometabolic risk profile in patients with psoriatic disease, the design of this study does not allow us to completely exclude such a possibility. Therefore, more (and better) studies are necessary in this regard.

## Figures and Tables

**Table 1:** Distribution of study variables stratified by sex.

Variables	Men, n: 309	Women, n: 263	Total, n: 572
Age (yrs), mean (SD)	47.5 (14.6)	45.7 (14.3)	46.7 (14.5)
Age at disease onset (yrs), median (min, max)	24.5 [1.00, 74.0]	20.0 [1.00, 78.0]	23.0 [1.00, 78.0]
Education level:			
Primary, n (%)	111 (35.9)	52 (19.8)	163 (28.5)
Secondary, n (%)	130 (42.1)	132 (50.2)	262 (45.8)
University, n (%)	68 (22.0)	79 (30.0)	147 (25.7)
Duration (yrs), mean (SD)	18.7 (14.3)	20.3 (15.4)	19.4 (14.8)
PsA, n (%)	81 (26.2)	90 (34.2)	171 (30)
Weight, mean (SD)	85.4 (14.6)	69.3 (13.9)	77.9 (16.4)
BMI, mean (SD)	28.5 (4.43)	26.7 (5.48)	27.6 (5.02)
Waist perimeter (cm), mean (SD)	101 (11.6)	91.4 (14.7)	96.7 (14.1)
PASI, mean (SD)	16.0 (11.9)	14.0 (12.0)	15.1 (12.0)
PASI ≥ 10, n (%)	174 (56.3)	120 (45.6%)	294 (51.4%)
Nail disease, n (%)	192 (62.1)	136 (51.7%)	328 (57.3%)
Plaque psoriasis, n (%)	281 (90.9)	216 (82.1)	497 (86.9)
Smoking, n (%)	99 (32.0)	97 (36.9)	196 (34.3)
Alcohol consumption (SDU), median (min, max)	0 [0, 2.00]	0 [0, 30.0]	0 [0, 30.0]
T1D, n (%)	11 (3.6)	11 (4.2)	22 (3.8)
T2D, n (%)	29 (9.4)	16 (6.1)	45 (7.9)
Hypertension, n (%)	68 (22.0)	46 (17.5)	114 (20.0)
Dyslipidemia, n (%)	68 (22.0)	45 (17.1)	113 (19.8)
NAFLD, n (%)	99 (32.0)	30 (11.4)	129 (22.6)
Patients with adverse coronary events, n (%)	20 (6.5)	13 (4.9)	33 (5.8)
Patients on systemic therapy, n (%)	203 (65.7)	167 (63.5)	370 (64.7)
Biologics, n (%)	125 (40.5)	108 (41.1)	233 (40.7)
Anti-TNF	63 (20.4)	55 (20.9)	118 (20.6)
Anti IL12/23	35 (11.3)	28 (10.6)	63 (11.0)
Anti-IL17	27 (8.8)	25 (9.5)	52 (9.1)

yrs: years; PsA: psoriatic arthritis; BMI: body mass index; PASI: psoriasis area and severity index; SDU: standard drink unit; T1D: type 1 diabetes; T2D: type 2 diabetes; NAFLD: non-alcoholic fatty liver disease; TNF: tumor necrosis factor; IL: interleukin.

**Table 2:** Distribution of study variables stratified by HLA-Cw6.

Variables	HLA-Cw6+, n: 241	HLA-Cw6−, n: 331
Age (yrs), mean (SD)	43.8 (13.7)	48.8 (14.7)
Age at disease onset (yrs), median (min, max)	18.0 [1.00, 78.0]	28.0 [1.00, 76.0]
Disease duration (yrs), mean (SD)	21.8 (14.8)	17.7 (14.5)
PsA, n (%)	65 (27.0)	106 (32.0)
Weight, mean (SD)	76.6 (15.3)	78.9 (17.1)
BMI, mean (SD)	27.2 (4.72)	28.0 (5.21)
Waist perimeter (cm), mean (SD)	95.3 (14.2)	97.7 (14.0)
PASI, mean (SD)	15.6 (10.9)	14.7 (12.7)
PASI ≥ 10, n (%)	134 (55.6)	160 (48.3)
Nail disease, n (%)	117 (48.5)	211 (63.7)
Plaque psoriasis, n (%)	219 (90.9)	278 (84.0)
Smoking, n (%)	87 (36.1)	109 (32.9)
Alcohol consumption (SDU), median (min, max)	0 [0, 30.0]	0 [0, 2.00]
T1D, n (%)	5 (2.1)	17 (5.1)
T2D, n (%)	10 (4.1)	35 (10.6)
Hypertension, n (%)	34 (14.1)	80 (24.2)
Dyslipidemia, n (%)	41 (17.0)	72 (21.8)
NAFLD, n (%)	54 (22.4)	75 (22.7)
Patients with adverse coronary events, n (%)	10 (4.1)	23 (6.9)
Patients on systemic therapy, n (%)	166 (68.9)	204 (61.6)
Biologics, n (%)	96 (39.8)	137 (41.4)
Anti-TNF	50 (20.7)	68 (20.5)
Anti-IL12/23	25 (10.4)	38 (11.5)
Anti-IL17	21 (8.7)	31 (9.4)

yrs: years; PsA: psoriatic arthritis; BMI: body mass index; PASI: psoriasis area and severity index; SDU: standard drink unit; T1D: type 1 diabetes; T2D: type 2 diabetes; NAFLD: non-alcoholic fatty liver disease; TNF: tumor necrosis factor; IL: interleukin.

## Data Availability

The materials and raw data described in the manuscript will be freely available to any researcher without breaching any participant’s confidentiality. To facilitate the revision of the results by other researchers, a file with the patient data is available as an excel file upon request to the corresponding author.
